# Prediction of Postoperative Pain and Side Effects of Patient-Controlled Analgesia in Pediatric Orthopedic Patients Using Machine Learning: A Retrospective Study

**DOI:** 10.3390/jcm14051459

**Published:** 2025-02-21

**Authors:** Young-Eun Joe, Nayoung Ha, Woojoo Lee, Hyo-Jin Byon

**Affiliations:** 1 Department of Anesthesiology and Pain Medicine, Asan Medical Center, University of Ulsan College of Medicine, Seoul 05505, Republic of Korea; 2 Department of Public Health Sciences, Graduate School of Public Health, Seoul National University, Seoul 08826, Republic of Korea; 3 Department of Anesthesiology and Pain Medicine, Anesthesia and Pain Research Institute, Yonsei University College of Medicine, Seoul 03722, Republic of Korea

**Keywords:** machine learning, pediatrics, orthopedic surgery, patient-controlled analgesia, postoperative pain management

## Abstract

**Background/Objectives**: Appropriate postoperative management, especially in pediatric patients, can be challenging for anesthesiologists. This retrospective study used machine learning to investigate the effects and complications of patient-controlled analgesia (PCA) in children undergoing orthopedic surgery. **Methods**: The medical records of children who underwent orthopedic surgery in a single tertiary hospital and received intravenous and epidural PCA were analyzed. Predictive models were developed using machine learning, and various demographic, anesthetic, and surgical factors were investigated to predict postoperative pain and complications associated with PCA. **Results**: Data from 1968 children were analyzed. Extreme gradient boosting effectively predicted moderate postoperative pain for the 6–24-h (area under curve (AUC): 0.85, accuracy (ACC): 0.79) and 24–48-h (AUC: 0.89, ACC: 0.87) periods after surgery. The factors that predicted moderate postoperative pain included the pain score immediately before the measurement period, the total amount of opioid infused, and age. For predicting side effects during the 6–24-h period after surgery, a least absolute shrinkage and selection operator model (AUC: 0.75, ACC: 0.64) was selected, while a random forest model (AUC: 0.91, ACC: 0.87) was chosen for the 24–48-h period post-surgery. The factors that predicted complications included the occurrence of side effects immediately before the measurement period, the total amount of opioid infused before the measurement period, and age. **Conclusions:** This retrospective study introduces machine-learning-based models and factors aimed at forecasting moderate postoperative pain and complications of PCA in children undergoing orthopedic surgery. This research has the potential to enhance postoperative pain management strategies for children.

## 1. Introduction

Postoperative pain management for pediatric patients is challenging due to various factors, including limited experience, insufficient assessment tools, variations in pain perception and analgesic needs among individuals, differences in pharmacokinetics and pharmacology between children and adults, and constraints in administering analgesics to children [1]. Inadequate pain management in pediatric patients can lead to sleep disturbances and altered pain responses, potentially contributing to the development of chronic pain [2,3]. Managing postoperative pain in pediatric patients following orthopedic surgery remains problematic [4], with concerns regarding the potential over-prescription of opioids in patients with poorly managed pain [5]. Moreover, individual variability exists in opioid responses [6,7], and effective communication can be challenging in pediatric patients undergoing scoliosis surgery. As a result, PCA often fails to provide adequate analgesia for relieving postoperative pain in children. Moreover, the utilization of PCA in children may be restricted due to apprehensions, such as respiratory depression, excessive sedation, programming errors, patient monitoring challenges, and PCA administration by proxy.

Patient-controlled analgesia (PCA) has been applied to manage postoperative pain in pediatric patients [8,9,10,11,12]. Nevertheless, PCA frequently falls short of delivering adequate analgesia to alleviate postoperative pain in children. The utilization of PCA in children may be restricted due to apprehensions, such as respiratory depression, excessive sedation, programming errors, patient monitoring challenges, and PCA administration by proxy [13]. By predicting the effects of PCA and identifying factors associated with its side effects, PCA can be administered more effectively and safely in pediatric patients undergoing surgery.

Machine learning (ML) holds promise in influencing various aspects of anesthesiology practice, spanning from perioperative assistance to critical care provision and outpatient pain management [14]. ML is employed for prognostic forecasting in pediatric medical domains, particularly in risk assessment among patients in intensive care units [15,16,17]. To our knowledge, there have been no investigations on the application of ML in assessing the effects of PCA for postoperative pain management in pediatric patients.

In pediatric patients, as in adults [18], preoperative risk stratification is crucial. This process necessitates the identification of various risk factors. Therefore, this study aimed to develop an ML-based model that is capable of predicting the effects and complications of PCA and investigating the factors influencing PCA in children undergoing orthopedic surgery.

## 2. Materials and Methods

### 2.1. Study Design and Ethics

This study was conducted on patients under 12 years of age who used intravenous PCA following pediatric orthopedic surgery from January 2013 to June 2021. The inclusion criteria involved patients with complete PCA records available for up to 48 h after surgery. Data from patients with height or weight exceeding 2 standard deviations and those who underwent multiple surgeries were excluded from the analysis. Raw PCA data were collected from the electronic medical record system using the Severance Clinical Research Analysis Portal.

This retrospective study was approved by the Institutional Review Board of the Yonsei University Health System, Seoul, Republic of Korea (4-2021-1185, date of approval: 18 October 2021), and the requirement for patient consent was waived.

### 2.2. Anesthetic Management and PCA Composition

General anesthesia was administered according to the standard institutional protocol. Standard monitoring, including pulse oximetry, electrocardiography, and non-invasive blood pressure measurement, was performed upon the arrival of the patient in the operating room. Anesthesia was induced using propofol (2 mg/kg) and rocuronium (0.6 mg/kg) and maintained using sevoflurane. Depending on the type of surgery, a regional block was performed when necessary.

The intravenous PCA regimen included 10–15 mcg/kg of fentanyl mixed with 100 mL of 0.9% normal saline, based on the surgery type and patient’s age. The basal rate was set at 2 mL per hour, the bolus analgesia for breakthrough pain was set at 0.5 mL, and the lock-out time was set to 15 min. PCA was administered 30 min before the end of the surgery.

The epidural PCA regimen included 0.15% ropivacaine mixed with 500 mL of 0.9% normal saline. The basal rate was set at 0.15 mL/kg per hour (maximum of 6 mL), the bolus analgesia for breakthrough pain was set at 0.1 mL/kg (maximum of 4 mL), and the lockout time was set to 30 min.

### 2.3. Variables and Outcome Measures

The covariates used in the ML model are provided in Table 1. The variables were divided into patient-, surgery-, anesthesia-, and postoperative-related factors.

We considered four types of binary outcome variables: moderate pain, severe pain, side effects, and nausea/vomiting (N/V). Moderate pain was defined as a pain score of 4–7, and severe pain was defined as a pain score of 7 or more. The Face-Legs-Activity-Cry-Consolability Scale was used for pediatric patients aged 3 years or younger, and the Wong–Baker Faces Scale was used for pediatric patients aged 4 years or older. The side effects evaluated included N/V, oversedation, dizziness, urinary incontinence, headache, pruritis, and respiratory depression. If any side effects occurred during any PCA period, it was classified as an occurrence. The severity of nausea was rated on a scale of 1 to 3, with mild nausea defined as 1, nausea requiring medication as 2, and nausea with vomiting as 3 points. N/V of 2 points or more was considered a side effect. These four outcome variables were measured 6–24 h and 24–48 h after orthopedic surgery, resulting in eight outcome variables for the analysis.

### 2.4. Statistical Analysis

#### 2.4.1. Machine Learning Methods and Model Selection

We considered the following six ML methods [19] to predict postoperative pain and side effects of PCA after surgery: (i) penalized logistic regression models with least absolute shrinkage and selection operator (LASSO) and ridge penalties, developed using the glmnet R package [20]; (ii) support vector machines (SVMs) with linear and radial basis kernels, created using the kernlab R package [21]; and (iii) random forest (RF) and extreme gradient boosting (XGB), performed using the randomForest and xgboost R packages [22,23]. Each method has tuning parameters (or hyperparameters), and the optimal tuning parameters were selected by stratified five-fold cross-validation (CV) using grid search over various combinations of the hyperparameters that maximized the performance metrics, as shown in Supplementary Table S1. The stratified five-fold CV approach ensures that the proportion of events for each fold is approximately the same, which is recommended for the imbalanced binary outcome variables [24].

The cross-validated area under the curve (AUC) and the cross-validated accuracy (ACC) are computed to evaluate the prediction performance of the six ML methods. We aggregated the predicted outcomes from each hold-out dataset and calculated a single cross-validated AUC value using all the predicted and the observed outcomes simultaneously [25]. The method showing the maximum AUC and ACC was selected as the best result.

#### 2.4.2. Variable Importance

For each outcome, we identified the top 10 most important features from the final model fitted to the full dataset with the optimal tuning parameter. We used the rminer package [26] to measure variable importance for the six ML methods. To explain how the variable importance of $x_{1}$ is computed, consider the final prediction model $\hat{f}(x)$, where $x=(x_{1},x_{2},\ldots ,x_{p}$) are p-dimensional features. We calculated the average absolute deviation (AAD) for $x_{1}$ as [26]:

$$AAD\left(x_{1}\right)=\frac{1}{7}\sum_{j=1}^{7}\left|\hat{f}\left(x_{1}^{j},\bar{x_{-1}}\right)-\underset{k}{Median}\{\hat{f}\left(x_{1}^{k},\bar{x_{-1}}\right)\}\right|$$

where $\bar{x_{-1}}=\left(\bar{x_{2}},\ldots ,\bar{x_{p}}\right)$ denotes the mean values for all except $x_{1}$. A similar formula applies to other inputs. To make these values lie within [0, 1] for convenience, we reported $r_{a}=AAD\left(x_{a}\right)/\sum_{a=1}^{p}AAD\left(x_{a}\right)$ as the variable importance for $x_{a}$.

#### 2.4.3. Descriptive Analysis

Continuous variables are expressed as mean ± standard deviation (SD) or as median (interquartile range) and categorical variables as number (percentage). A *p*-value of <0.05 was considered statistically significant. All statistical analyses were performed using R version 4.3.3 (https://www.r-project.org/).

## 3. Results

### 3.1. Patient Characteristics

**Figure 1:**
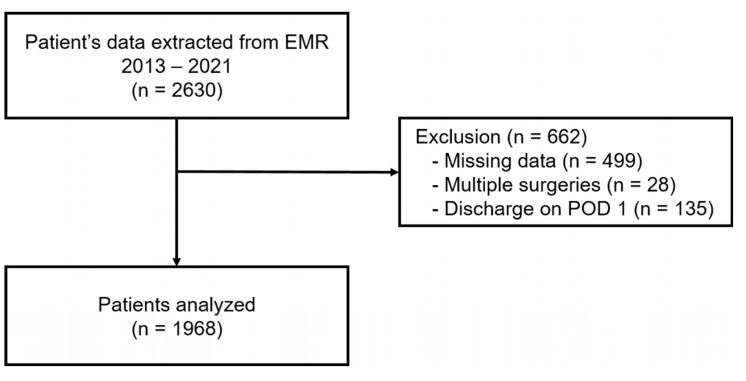
Flow sheet of patient enrollment. EMR, electronic medical record; POD, postoperative day.

The medical records of 2630 patients were obtained from the electronic medical record system and included in this study. However, data from 662 patients were excluded due to missing values, multiple surgeries during a single hospitalization, or discharge on postoperative day 1. Thus, data from a total of 1968 patients aged less than 12 years who underwent orthopedic surgery between 2013 and 2021 were analyzed in this study (Figure 1). The baseline characteristics of all included patients are detailed in Table 2.

### 3.2. Outcome Measures

#### 3.2.1. Pain Score

Table 3 describes the average pain scores, postoperative pain severity, and occurrence of side effects for each time interval. Within 6–24 h after surgery, 356 patients (18.1%) reported moderate to severe pain, and among them, 50 patients (2.5%) experienced severe pain. Between 24 and 48 h after surgery, 100 patients (10.6%) experienced moderate to severe pain, and 10 patients (1.1%) reported severe pain.

Among the six models assessed, those with high AUC and ACC values were selected. Table 4 shows the top three prediction performances of each ML model. The XGB model emerged as the best performer in predicting moderate pain (AUC: 0.85, ACC: 0.79) and severe pain (AUC: 0.88, ACC: 0.76) during the 6–24-h post-surgery periods. The LASSO model was chosen for predicting moderate pain (AUC 0.89, ACC 0.87) and severe pain (AUC 0.98, ACC 0.96) during 24–48-h periods after surgery.

**Figure 2:**
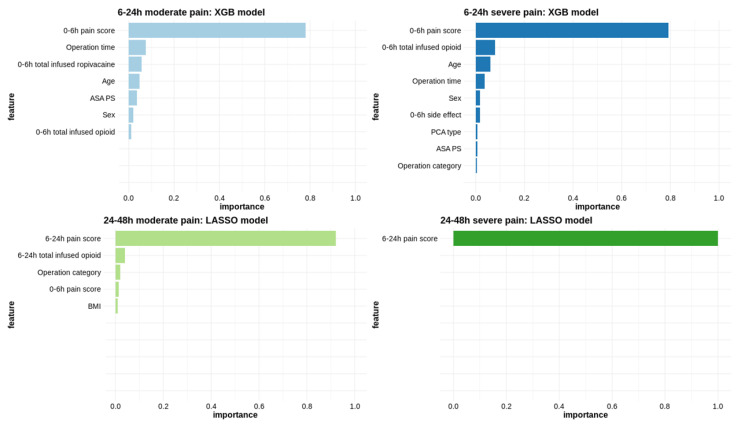
Variable importance in predicting moderate and severe pain. ASA PS, American Society of Anesthesiologists physical status; BMI, body mass index; LASSO, least absolute shrinkage and selection operator; PCA, patient-controlled analgesia; XGB, extreme gradient boosting.

Variables such as the pain score immediately before the measurement period, total opioid amount until the measurement period, operation time, and patient age were associated with both moderate to severe pain and severe pain within 6–24 h after surgery (Figure 2). The most important variable for predicting moderate and severe pain within 24–48 h post-surgery was the pain score recorded during the 6–24-h period.

#### 3.2.2. Side Effects

The overall occurrence rate of side effects was 16.5%. Specifically, within 6–24 h after surgery, the incidence of side effects was 6.1% (121 patients), and within 24–48 h after surgery, it was 1.7% (33 patients). Nausea and vomiting, a significant side effect, were observed in 90 patients (4.6%) within 6–24 h after surgery and in 23 patients (1.2%) within 24–48 h after surgery (Table 3).

The LASSO model (AUC: 0.75, ACC: 0.64) and the RF model (AUC: 0.91, ACC: 0.87) emerged as the top-performing models for predicting side effects during the 6–24-h and the 24–48-h postoperative periods, respectively. Regarding the prediction of nausea and vomiting, the most prevalent side effect, the Ridge model (AUC: 0.72, ACC: 0.77) was selected for the 6–24-h postoperative period, while the XGB model (AUC: 0.91, ACC: 0.81) was chosen for the 24–48-h postoperative period (Table 4).

For the prediction performances by age (6 years and under, over 6 years), the younger subgroup showed slightly higher AUC and ACC compared to the older subgroup. Overall, both subgroups performed similarly to the whole sample. The details about the top three prediction results are provided in Supplementary Table S5.

**Figure 3:**
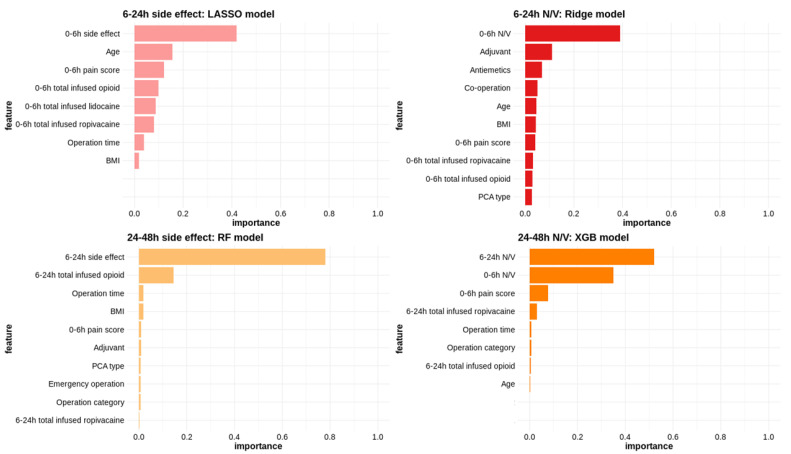
Variable importance in predicting side effects and nausea/vomiting. BMI, body mass index; LASSO, least absolute shrinkage and selection operator; N/V, nausea/vomiting; PCA, patient-controlled analgesia; RF, random forest; XGB, extreme gradient boosting.

The main predictors of side effects included the occurrence of side effects immediately before the measurement period, total opioid infusion amount, and patient age. Additionally, the most significant variable for predicting N/V after surgery was the presence of N/V immediately before the measurement period (Figure 3).

## 4. Discussion

Our study aimed to predict postoperative pain and side effects in pediatric patients using PCA following orthopedic surgery. We developed an ML-based model that was capable of predicting moderate and severe pain for up to 48 h after surgery. We assessed six ML models, including RF, SVM, XGB, LASSO, and Ridge, and selected those with the highest AUC and ACC values. The chosen models had AUC and ACC values exceeding 0.8 [27], indicating their effectiveness in predicting postoperative pain and side effects. Postoperative pain and adverse effects are influenced by a wide range of factors, including patient characteristics, anesthesia, and surgical variables. Therefore, predicting these outcomes is inherently challenging. Considering these complexities, our models can be evaluated as having strong predictive performance. By applying this model to various patient variables, we can better anticipate and proactively manage postoperative pain and side effects associated with PCA. Additionally, among the predictive factors, prior occurrences of pain and side effects had the highest variable importance, highlighting the need for more active intervention by clinicians. The models predicting pain and side effects varied at each time point. This finding suggests the need to develop an appropriate predictive model tailored to the timing of pain and side effects. Furthermore, ML-based models could be beneficial in predicting pain and preventing the side effects of PCA. For patients expected to experience pain or side effects, a machine learning model can be utilized to predict risk stratification before surgery, allowing for adjustments to the PCA regimen accordingly.

Various variables were used to develop the model for predicting postoperative pain, and the importance of each variable was verified (Figure 2). The main variables that predicted postoperative pain were the pain score during the previous time period, age, sex, BMI, ASA classification, and the total amount of opioids infused. Among them, the ASA classification and BMI are variables identified for the first time in this study [28]. In patients undergoing orthopedic surgeries such as scoliosis correction, severe preoperative pain is associated with increased postoperative pain [29], and more than 50% of patients experience persistent pain after surgery [30,31]. Considering the findings of this study together, proactive pain management before, during, and after surgery is essential. By incorporating these variables into pain prevention and treatment strategies following orthopedic surgery, the outcomes of pediatric patients could be improved.

We also identified factors associated with the side effects of PCA through ML-based modeling. Similar to the model predicting pain, factors such as prior occurrences of side effects, age, BMI, and the type of surgery were associated with the likelihood of side effects. In patients with these clinical risk factors, the amount of antiemetics mixed in PCA should be increased, or the patient should be closely monitored for the need for additional antiemetics and managed accordingly.

Our study has several limitations. First, our study did not consider the pain score and side effects between immediately after surgery and 6 h after surgery. The primary objective of this study was to develop a model to predict the effects and side effects of PCA. Therefore, the decision was made to exclude data from PCA administered 30 min before the end of the surgery, as this period was deemed insufficient to achieve adequate plasma concentrations. Instead, this study focused on evaluating postoperative pain and side effects during the 6–24-h and 24–48-h periods after surgery. However, as pain is most severe immediately after surgery (Table 3), future research is needed to develop predictive models targeting pain and side effects during this immediate postoperative period. Second, our study is retrospective and used data from a single tertiary center. It evaluated a heterogeneous cohort comprising various surgical types and a wide range of pediatric age groups. Additionally, there were no significant variations in the dose of the PCA regimen or the rescue medication administered. Prospective, large-scale, multicenter trials are needed to enhance postoperative pain control in pediatric patients. Third, the class imbalance (e.g., 24–48 h severe pain events) problem is a potential limitation. Although we used a stratified 5-fold CV to address imbalanced binary outcomes, the rare event of severe pain may still affect the model’s performance. Consequently, it is necessary to interpret the results for severe pain during the 24–48 h period carefully. Further research is required to explore more effective methods for dealing with rare events.

## 5. Conclusions

This retrospective study demonstrated that ML-based models effectively predicted postoperative pain and side effects in children using PCA after orthopedic surgery. The use of ML can potentially improve postoperative pain management in pediatric patients.

## Figures and Tables

**Table 1:** Covariates used in machine learning models.

Patient-related	Anesthesia-related
Age	Type of PCA (IV, epidural)
Sex	PCA-related
Height	Total amount of opioid infused (mcg)
Weight	Adjuvant pain killer mixed
BMI (categorical: <18.5, 18.5–25, ≥25)	Anti-emetics mixed
ASA PS	
Surgery-related	Post-operative
Operation time	Pain score in PACU
Complexity of surgery	Pain score 0–6 h postoperatively
Co-operation	Pain score 6–24 h postoperatively
Emergency operation	Analgesia used in ward

ASA PS, American Society of Anesthesiologists physical status; BMI, body mass index; PACU, post-anesthetic care unit; PCA, patient-controlled analgesia.

**Table 2:** Baseline characteristics of patients.

Variable	*n* = 1968
Age (years)	8.06 ± 3.16
Female	893 (45.4)
Height (cm)	126.68 ± 21.87
Weight (kg)	30.84 ± 14.87
BMI (kg/m^2^)	
<18	1234 (62.7)
18–25	611 (31.0)
≥25	123 (6.2)
ASA PS	
1	869 (44.2)
2	928 (47.2)
3	171 (8.7)
Operation time (min)	154.75 ± 113.73
Anesthesia time (min)	174.63 ± 129.26

Data are expressed as the mean ± standard deviation or count (%), as appropriate. ASA PS, American Society of Anesthesiologists physical status; BMI, body mass index.

**Table 3:** Postoperative pain score and incidence of side effects.

	Overall	Sex Group			Age Group			
		Men	Women	*p*	Age ≤ 1	1 < Age ≤ 6	Age > 6	*p*
*n*	1968	1075	893		81	503	1384	
Postoperative period 0–6 h								
Pain score	2.33 ± 2.44	2.33 ± 2.39	2.34 ± 2.49	0.939	0.70 ± 1.57	1.44 ± 2.03	2.75 ± 2.49	<0.001
Severity of pain				0.283				<0.001
Mild (<4)	1384 (70.3)	765 (71.2)	619 (69.3)		77 (95.1)	415 (82.5)	892 (64.5)	
Moderate (≥4, <7)	466 (23.7)	254 (23.6)	212 (23.7)		3 (3.7)	78 (15.5)	385 (27.8)	
Severe (≥7)	117 (5.9)	55 (5.1)	62 (6.9)		1 (1.2)	10 (2.0)	106 (7.7)	
Any side effect	172 (8.7)	102 (9.5)	70 (7.8)	0.226	3 (3.7)	29 (5.8)	140 (10.1)	0.003
Nausea/vomiting	121 (6.1)	69 (6.4)	52 (5.8)	0.65	3 (3.7)	18 (3.6)	100 (7.2)	0.009
Postoperative period 6–24 h								
Pain score	1.58 ± 2.39	1.48 ± 1.97	1.69 ± 2.81	0.05	0.41 ± 1.51	0.96 ± 2.96	1.87 ± 2.13	<0.001
Severity of pain				0.244				<0.001
Mild (<4)	1607 (81.7)	894 (83.2)	713 (79.8)		78 (96.3)	453 (90.1)	1076 (77.7)	
Moderate (≥4, <7)	306 (15.5)	155 (14.4)	151 (16.9)		0 (0.0)	39 (7.8)	267 (19.3)	
Severe (≥7)	50 (2.5)	23 (2.1)	27 (3.0)		3 (3.7)	9 (1.8)	38 (2.7)	
Any side effect	121 (6.1)	71 (6.6)	50 (5.6)	0.406	1 (1.2)	10 (2.0)	110 (7.9)	<0.001
Nausea/vomiting	90 (4.6)	50 (4.7)	40 (4.5)	0.942	1 (1.2)	9 (1.8)	80 (5.8)	<0.001
Postoperative period 24–48 h								
Pain score	0.99 ± 1.69	1.02 ± 1.74	0.96 ± 1.64	0.558	0.35 ± 1.35	0.48 ± 1.26	1.21 ± 1.80	<0.001
Severity of pain				0.125				0.023
Mild (<4)	846 (43.0)	448 (41.7)	398 (44.6)		38 (46.9)	226 (44.9)	582 (42.1)	
Moderate (≥4, <7)	90 (4.6)	42 (3.9)	48 (5.4)		1 (1.2)	11 (2.2)	78 (5.6)	
Severe (≥7)	10 (0.5)	7 (0.7)	3 (0.3)		1 (1.2)	1 (0.2)	8 (0.6)	
Any side effect	33 (1.7)	21 (2.0)	12 (1.3)	0.383	0 (0.0)	5 (1.0)	28 (2.0)	0.149
Nausea/vomiting	23 (1.2)	13 (1.2)	10 (1.1)	1	0 (0.0)	3 (0.6)	20 (1.4)	0.192

Data are expressed as the mean ± standard deviation or count (%), as appropriate.

**Table 4:** Top three prediction results of each machine learning model for eight outcomes.

		Specificity	Sensitivity	ACC	AUC
Moderate pain 6–24 h	XGB	0.79	0.79	0.79	0.85
	LASSO	0.87	0.67	0.83	0.84
	RF	0.85	0.7	0.83	0.84
Severe pain 6–24 h	XGB	0.75	0.88	0.76	0.88
	RF	0.79	0.76	0.79	0.85
	LASSO	0.92	0.64	0.91	0.83
Moderate pain 24–48 h	LASSO	0.87	0.88	0.87	0.89
	Ridge	0.8	0.89	0.81	0.89
	XGB	0.85	0.89	0.86	0.88
Severe pain 24–48 h	LASSO	0.96	1	0.96	0.98
	XGB	0.93	1	0.93	0.98
	RF	0.94	0.9	0.94	0.96
Side effect 6–24 h	LASSO	0.63	0.74	0.64	0.75
	XGB	0.66	0.71	0.66	0.74
	Ridge	0.63	0.74	0.64	0.74
Side effect 24–48 h	RF	0.87	0.82	0.87	0.91
	Ridge	0.86	0.88	0.86	0.9
	XGB	0.8	0.91	0.8	0.89
Nausea/vomiting 6–24 h	Ridge	0.78	0.52	0.77	0.72
	XGB	0.5	0.86	0.52	0.72
	LASSO	0.38	0.92	0.4	0.72
Nausea/vomiting 24–48 h	XGB	0.81	0.87	0.81	0.91
	Ridge	0.9	0.83	0.9	0.89
	RF	0.78	0.78	0.78	0.85

AUC, cross-validated area under the curve; ACC, cross-validated accuracy; LASSO, least absolute shrinkage and selection operator; RF, random forest; XGB, extreme gradient boosting.

## Data Availability

The data presented in this study are available on request from the corresponding author. The data are not publicly available to ensure confidentiality.

## Supplementary Materials

The following supporting information can be downloaded at: https://www.mdpi.com/article/10.3390/jcm14051459/s1, Table S1: Hyperparameter settings for the six machine learning methods; Table S2: Categories of surgical procedure; Table S3: Covariates according to pain score during postoperative 6–24 h; Table S4: Covariates according to pain score during postoperative 24–48 h; Table S5: Subgroup analysis by age group.
