# Supplementary material for: Prediction of Postoperative Pain and Side Effects of Patient-Controlled Analgesia in Pediatric Orthopedic Patients Using Machine Learning: A Retrospective Study
Source: J Clin Med. 2025 Feb 21;14(5):1459. doi: 10.3390/jcm14051459 (PMC11899821; doi:10.3390/jcm14051459)
Supplement: Supplementary file 1 [file jcm-14-01459-s001.zip › jcm-3423003-supplementary.pdf]

Supplementary Table S1. Hyperparameter settings for the six machine learning methods

Algorithm	Hyperparameters
Ridge	alpha = 0, lambda = exp(seq(-6,6,length=100))
LASSO	alpha = 1, lambda = exp(seq(-6,6,length=100))
SVM (linear)	Kernel=" vanilladot", Cost = exp(-3:3)
SVM (radial)	Kernel=" rbfdot", Cost = exp(-8:3), sigma=exp(-5:3)
RF	ntree=c(50,100,300,500,1000,2000),<br>mtry=c(floor(sqrt(p))-2, floor(sqrt(p))-1, floor(sqrt(p)), floor(p/3)),<br>where p is the number of columns
XGB	eta=c(0.05, 0.01,0.1,0.3, 0.5),<br>max_depth=c(3,6,8,10),<br>gamma=c(0,0.3,0.5,1,3,5),<br>min_child_weight=c(1,3,5)

LASSO, least absolute shrinkage and selection operator; RF, random forest; SVM, support vector machines; XGB, extreme gradient boosting.

Supplementary Table S2. Categories of surgical procedure

Operation category (n, %)	n = 1968
0<br>A1 pulley release, tenotomy, Adhesiolysis, Arthrogram, Arthroscopic surgery, Aseptic dressing, Closed pinning, Closed reduction and internal fixation, Debridement and curettage, Excision, Fasciotomy, Hardware removal, Ligament reconstruction, Meniscal repair, Neurolysis, Tendon lengthening	500 (25.4)
1<br>ACL reconstruction, Arthrotomy, Bone graft, Capsulorrhaphy, Capsulotomy, Curettage of bone, Division of syndactyly, Epiphysiodesis, External fixation, Open reduction and internal fixation, Osteosynthesis	623 (31.7)
2<br>Arthrolysis, Above knee amputation, Below knee amputation, Limb lengthening procedures, Mechanical correction for scoliosis, Ostectomy, Osteotomy, Patellectomy, Posterior spinal fusion, Resection arthroplasty	845 (42.9)

Data are presented as count (%).

Supplementary Table S3. Covariates according to pain score during postoperative 6-24 h

**Variables**	**Mild (n=1607)**	**Moderate (n=306)**	**Severe (n=50)**	***P*-value**
**Age (year)**	7.83 (3.24)	9.23 (2.38)	8.42 (3.20)	<0.001
**Female**	713 (44.4)	151 (49.3)	27 (54.0)	0.128
**Height (cm)**	125.44 (22.24)	133.33 (18.58)	125.81 (22.18)	<0.001
**Weight (kg)**	7.83 (3.24)	9.23 (2.38)	8.42 (3.20)	<0.001
**BMI (kg/m^2)**				0.178
Low (<18.5)	1029 (64.0)	174 (56.9)	29 (58.0)	
Moderate	482 (30.0)	109 (35.6)	17 (34.0)	
Over (≥25)	96 (6.0)	23 (7.5)	4 (8.0)	
**ASA PS**				<0.001
1	736 (45.8)	117 (38.2)	15 (30.0)	
2	718 (44.7)	174 (56.9)	32 (64.0)	
3	153 (9.5)	15 (4.9)	3 (6.0)	
**Operation time (min)**	144.22 (109.07)	194.06 (119.16)	253.64 (125.67)	<0.001
**Anesthetic time (min)**	162.62 (123.96)	219.68 (134.80)	287.92 (142.68)	<0.001
**Pain score 0-6 h**		4.83 (2.53)	5.88 (3.19)	
**Overall side effect 0-6 h**	125 (7.8)	41 (13.4)	5 (10.0)	0.006
**Nausea/vomiting 0-6 h**	84 (5.2)	31 (10.1)	5 (10.0)	0.002

Data are presented as the mean ± standard deviation or count (%) as appropriate.

ASA PS, American Society of Anesthesiologists physical status; BMI, body mass index; PACU, post-anesthetic care unit.

Supplementary Table S4. Covariates according to pain score during postoperative 24-48 h

**Variables**	**Mild (n=846)**	**Moderate (n=90)**	**Severe (n=10)**	***P*-value**
**Age (year)**	7.92 (3.20)	9.14 (2.47)	8.20 (3.12)	0.002
**Female**	398 (47.0)	48 (53.3)	3 (30.0)	0.283
**Height (cm)**	124.36 (21.84)	132.05 (20.41)	121.34 (22.00)	0.005
**Weight (kg)**	29.44 (14.46)	33.80 (14.73)	28.99 (16.41)	0.025
**BMI (kg/m^2)**				0.026
Low (<18.5)	542 (64.1)	48 (53.3)	7 (70.0)	
Moderate	254 (30.0)	31 (34.4)	1 (10.0)	
Over (≥25)	50 (5.9)	11 (12.2)	2 (20.0)	
**ASA PS**				0.642
1	326 (38.5)	29 (32.2)	3 (30.0)	
2	412 (48.7)	51 (56.7)	6 (60.0)	
3	108 (12.8)	10 (11.1)	1 (10.0)	
**Operation time (min)**	186.19 (116.54)	223.32 (125.90)	270.90 (127.53)	0.002
**Anesthetic time (min)**	211.34 (133.82)	250.10 (141.38)	317.20 (151.62)	0.002
**Pain score 0-6 h**	2.24 (2.46)	4.81 (3.02)	6.20 (2.53)	<0.001
**Pain score 6-24 h**	1.29 (1.88)	4.84 (2.10)	7.90 (1.29)	<0.001
**Side effect 0-6 h**	64 (7.6)	8 (8.9)	0 (0.0)	0.596
**Side effect 6-24 h**	68 (8.0)	12 (13.3)	2 (20.0)	0.104
**Nausea/vomiting 0-6 h**	45 (5.3)	7 (7.8)	0 (0.0)	0.464

**Nausea/vomiting 6-24 h**	48 (5.7)	11 (12.2)	2 (20.0)	0.012

Data are presented as the mean ± standard deviation or count (%) as appropriate.

ASA PS, American Society of Anesthesiologists physical status; BMI, body mass index; PACU, post-anesthetic care unit.

Supplementary Table S5. Subgroup analysis by age group

(1) 0<Age≤6

	Algorithm	Specificity	Sensitivity	ACC	AUC
**Moderate pain 6–24 h**	XGB	0.8832	0.7255	0.8694	0.8591
	SVM radial	0.8456	0.7647	0.8385	0.8487
	Ridge	0.8682	0.7451	0.8574	0.8439
**Severe pain 6–24 h**	XGB	0.8895	0.8333	0.8883	0.9228
	SVM radial	0.7316	1	0.7371	0.9227
	RF	0.8386	0.8333	0.8385	0.9027
**Moderate pain 24–48 h**	RF	0.8788	1	0.8849	0.9609
	SVM radial	0.8258	1	0.8345	0.9351
	XGB	0.9432	0.9286	0.9424	0.9328
**Severe pain 24–48 h ***	-	NaN	NaN	NaN	NaN
	-	NaN	NaN	NaN	NaN
	-	NaN	NaN	NaN	NaN
**Side effect 6–24 h**	XGB	0.8063	0.7273	0.8048	0.7711
	Ridge	0.8202	0.6364	0.8168	0.7151
	LASSO	0.7749	0.6364	0.7723	0.7133
**Side effect 24–48 h**	RF	0.8995	1	0.9003	0.953
	Ridge	0.974	0.6	0.9708	0.8444
	XGB	0.9549	0.6	0.9519	0.8113
**Nausea/vomiting 6–24 h**	XGB	0.6986	0.8	0.7003	0.7209
	Ridge	0.6341	0.8	0.637	0.6923
	LASSO	0.8049	0.6	0.8014	0.6779
**Nausea/vomiting 24–48 h**	RF	0.9309	1	0.9313	0.9781
	SVM radial	0.8394	1	0.8402	0.8883
	LASSO	0.5527	1	0.555	0.7185

* The stratified 5-fold cross-validation failed due to the very low number of events in this subgroup.

AUC, cross-validated area under the curve; ACC, cross-validated accuracy; LASSO, least absolute shrinkage, and selection operator; RF, random forest; SVM, support vector machine; XGB, extreme gradient boosting

(2) 6<Age≤12

	Algorithm	Specificity	Sensitivity	ACC	AUC
**Moderate pain 6–24 h**	XGB	0.7965	0.7508	0.7864	0.833
	RF	0.7667	0.7803	0.7697	0.8232
	SVM linear	0.8485	0.6656	0.8081	0.8225
**Severe pain 6–24 h**	XGB	0.6456	0.8947	0.6524	0.8475
	LASSO	0.8764	0.6316	0.8697	0.8143
	RF	0.8206	0.6579	0.8161	0.8142
**Moderate pain 24–48 h**	LASSO	0.8436	0.8488	0.8443	0.8809
	XGB	0.8333	0.8721	0.8383	0.8747
	Ridge	0.8179	0.8023	0.8159	0.8691
**Severe pain 24–48 h**	LASSO	0.9394	1	0.9401	0.9797
	XGB	0.9152	1	0.9162	0.976
	Ridge	0.8864	1	0.8877	0.9544
**Side effect 6–24 h**	Ridge	0.5452	0.7818	0.564	0.7108
	LASSO	0.7266	0.5909	0.7158	0.71
	XGB	0.8397	0.4636	0.8098	0.698
**Side effect 24–48 h**	Ridge	0.7975	0.9643	0.8009	0.9221
	XGB	0.8574	0.8929	0.8581	0.9087
	RF	0.8795	0.8214	0.8783	0.9047
**Nausea/vomiting 6–24 h**	LASSO	0.5357	0.75	0.5481	0.6895
	Ridge	0.462	0.8125	0.4823	0.6837
	SVM linear	0.3561	0.9	0.3876	0.6605
**Nausea/vomiting 24–48 h**	Ridge	0.8295	0.95	0.8313	0.9311
	XGB	0.8494	0.9	0.8501	0.9247
	RF	0.8229	0.85	0.8233	0.8775

AUC, cross-validated area under the curve; ACC, cross-validated accuracy; LASSO, least absolute shrinkage, and selection operator; RF, random forest; SVM, support vector machine; XGB, extreme gradient boosting
